# Association between indigenous status and Body Mass Index (BMI) in Australian adults: Does sleep duration affect the relationship?

**DOI:** 10.1371/journal.pone.0263233

**Published:** 2022-02-16

**Authors:** Melissa Deacon-Crouch, Isabelle Skinner, Joseph Tucci, Steve Begg, Ruth Wallace, Timothy Skinner

**Affiliations:** 1 Rural Department of Nursing and Midwifery, La Trobe Rural Health School, La Trobe University, Bendigo, Victoria, Australia; 2 Department of Pharmacy and Biomedical Science, La Trobe Institute for Molecular Science, La Trobe University, Victoria, Australia; 3 Rural Department of Community Health, La Trobe Rural Health School, La Trobe University, Bendigo, Victoria, Australia; 4 College of Indigenous Futures, Education & the Arts, Charles Darwin University, Northern Territory, Australia; 5 Department of Psychology, University of Copenhagen, Copenhagen, Denmark; University of Western Australia, AUSTRALIA

## Abstract

**Background:**

Overweight/obesity is a well-defined risk factor for a variety of chronic cardiovascular and metabolic diseases. Sleep duration has been associated with overweight/obesity and other cardio metabolic and neurocognitive problems. Notably, overweight/obesity and many of the associated comorbidities are prevalent in Indigenous Australians. Generally, sleep duration has been associated with BMI for Australian adults but information about Australian Indigenous adults’ sleep is scant. A recent report established that sleep is a weak predictor of obesity for Indigenous Australian adults.

**Aim:**

To determine whether sleep remains a predictor of obesity when physical activity, diet and smoking status are accounted for; and to determine whether sleep duration plays a mediating role in the relationship between Indigenous status and BMI.

**Methods:**

Statistical analyses of 5,886 Australian adults: 5236 non-Indigenous and 650 Indigenous people aged over 18 years who participated in the Australian Health Survey 2011–2013. Demographic and lifestyle characteristics were described by χ^2^ and t-tests. ANOVA was used to determine the variables that significantly predicted BMI and sleep duration. Stepwise regression analyses were performed to determine the strongest significant predictors of BMI. Sleep duration was self-reported; BMI was calculated from measurement.

**Results:**

The study revealed two main findings: (i) short sleep duration was an independent predictor of obesity (adjusted-*R*^*2*^ = 0.056, *p* <0.0001); and (ii) controlling for sleep duration and other possible confounders, Indigenous status was a significant predictor of BMI overweight/obesity. Sleep duration played a weak, partial mediator role in this relationship. Increased BMI was associated with lower socioeconomic status and level of disadvantage of household locality for non-remote Indigenous and non-Indigenous people.

**Conclusion:**

Indigenous status strongly predicted increased BMI. The effect was not mediated by the socioeconomic indicators but was partially mediated by sleep duration.

## Introduction

Overweight/obesity is a well-established risk factor for the development of chronic illnesses including cardio-metabolic diseases and cancer [1, 2], with considerable contribution to the total burden of disease [3] and hence, the economic costs to the community [4]. Prior to Western colonisation, Indigenous Australians led traditional lifestyles that promoted lean body weight [5]. However the incidence of overweight/obesity in Indigenous Australians is now widespread, with 71% of adults assessed as overweight (27%) or obese (44%) [6]. The high prevalence of obesity and associated chronic illnesses experienced by Indigenous Australians is a complex and multifactorial phenomenon [7] related to colonisation with multiple socioeconomic and behavioural dimensions [8] including political marginalisation and ingrained institutional, structural and interpersonal racial prejudice [9]. To address this pervasive situation, Australian Indigenous peoples released the Uluru Statement from the Heart [10] seeking constitutional reforms to empower self-determination by giving Indigenous peoples a voice to impact laws and policies that affect them. To date, despite widespread Australian public support for self-determination of Indigenous Australians, constitutional change has not occurred [11].

Sleep duration has been shown to be an important health factor associated with obesity and other metabolic, immunologic, and cardiovascular diseases as well as psychiatric and other neurocognitive dysfunctions in non-Indigenous Australians [12]. Previous studies with children have shown that short sleep duration is an accepted predictor of obesity for children [13–15], including Indigenous Australian children [16]. Sleep duration has been shown to have a mediating role in the relationship between Indigenous status and body mass index (BMI) for Indigenous Australian school-aged children [7].

Associations between sleep duration and BMI in the general Australian adult population (including Indigenous people) have been reported [17, 18], however to date, information about sleep duration specific to Indigenous Australians remains scant [19]. Lack of sleep has previously been identified as an issue of concern by one group of Australian Indigenous people [20] and our recent study of Australian adults showed that less than 7 hours sleep duration predicted obesity for both Indigenous and non-Indigenous adults [21]. However, it remains unknown if differences in sleep duration account for any of the variation in BMI between Indigenous and non-Indigenous Australian adults.

Sleep duration has been associated with both chronic illness and overweight/obesity in other populations [22] yet there is little known as to whether sleep duration is associated with these conditions for Indigenous Australian adults. If sleep duration is found to influence the risk of obesity and by extension, risk of chronic illness for Indigenous people, interventions to help optimise sleep duration may improve health outcomes.

Lifestyle factors such as diet, physical activity and smoking that are associated with sleep and BMI were also considered in this study [23]. Diet and sleep appear to have a bi-directional relationship: sleep has been reported to affect eating behaviours [24] and metabolic processes [25] while diet, particularly fruit and vegetable intake, has been shown to influence sleep efficiency and duration [26]. A recent meta-analysis revealed that physical activity significantly improved both subjective perceptions and objective measures of sleep in healthy individuals [27]. Conversely, lack of physical activity has been associated with abdominal obesity and increased risks of cardiometabolic diseases [28] and physical activity has been endorsed in Australia to help reduce obesity and the risks for other cardiometabolic diseases [3]. Smoking status has also been found to influence both BMI [29, 30] and sleep [31, 32].

Having previously established that sleep is a weak predictor of obesity for Indigenous Australian adults, the aim of this study is to determine (i) whether sleep remains a predictor of obesity when selected lifestyle variables are accounted for; and (ii) whether sleep duration plays a mediating role in the relationship between Indigenous status and BMI.

## Methods

This study used the same dataset and followed on from our previous report [21]. In short, the dataset from participants in the Australian Health Survey (AHS) and the complementary Australian Aboriginal and Torres Strait Islander Health Survey (AATSIHS) was provided by the Australian Bureau of Statistics (ABS). All data were de-identified and protected by the secrecy provisions in the Australian Government *Census and Statistical Act 1905*, rendering it impossible to identify individual respondents from published findings. In consultation with Aboriginal and Torres Strait Islander authorities (National Aboriginal and Torres Strait Islander Health Equality Council; National Aboriginal Community Controlled Health Organisation; National Advisory Group on Aboriginal and Torres Strait Islander Health Information and Data; Office for Aboriginal and Torres Strait Islander Health) and other stakeholders, the ABS was granted ethical approval by the Australian Government Department of Health and Ageing’s Departmental Ethics Committee for the AATSIHS survey to be administered to the Aboriginal and Torres Strait Islander participants in October 2011 and for the AHS to be administered to the general Australian public (which may include Indigenous people) in March 2011 [33, 34]. Complete details about the AHS and AATSIHS structure and design, ethics approval and sampling methods and data collection are described elsewhere [35, 36]. The AHS and AATSIHS comprised a number of corresponding components including the National Nutrition and Physical Activity Survey (NNPAS) 2011–2013 [37] and the matching National Aboriginal and Torres Strait Islander Nutrition and Physical Activity Survey (NATSINPAS) [38]. The 5886 participants in this study included 5236 non-Indigenous adults who participated in the NNPAS and 650 Indigenous adults who participated in the NATSINPAS in the period 2012–2013. The participant age range was 18–75 years. Following ABS protocols, authorisation to access data from the Confidentialised Unit Record Files (CURF) from the NNPAS and the NATSINPAS was gained from the ABS in January 2017. Survey questions in the NATSINPAS were based on those from the NNPAS and so were directly comparable. All data were accessed, analysed, and cleared for research use through an online Australian Bureau of Statistics (ABS) DataLab virtual desktop portal. The secondary analysis of ABS data did not require separate University Human Research Ethics approval [39]. In this study the term Indigenous refers to all persons who identified themselves as being of Aboriginal, Torres Strait Islander, or both Aboriginal and Torres Strait Islander origin. Non-Indigenous refers to all other participants. Area of residence of participants was classified according to the Australian Statistical Geographic Standard (ASGS) which identifies locations based on the relative access to services for people living in these areas. Locations are defined as Major Cities (ASGS 1), Inner Regional (ASGS 2), Outer Regional (ASGS 3), Remote (ASGS 4) or Very Remote (ASGS 5) [40]. In this study the term non-remote refers to the urban and regional areas of Australia (ASGS1, ASGS2, ASGS3) that have been assessed as having relatively good access to services. Remote (ASGS4) and very remote (ASGS 5) areas of Australia have little or very limited access to services. The ABS released data for non-remote NATSINPAS participants only, hence analysis of data in this study pertains only to those Indigenous and non-Indigenous peoples who resided in non-remote locations (ASGS 1, 2 & 3).

### Measures

As a follow on from the previous study, the same measures were employed [21] with the addition of lifestyle variables related to diet, physical activity and smoking status. In short, **sleep duration** was subjectively reported and declared as a typical night’s sleep. Those who reported an atypical night’s sleep were excluded from the analysis. A typical night’s sleep was considered to reflect the approximate pattern and/or number of hours that occurred on that same night of the week over time. Sleep duration was stratified into 3 categories: Short sleep (<7 h), optimal sleep (7–9 h) and long sleep (>9 h) [41]; **BMI**: derived from height and weight objectively measured by the interviewers. **Index of Relative Socioeconomic Disadvantage 2011 (SEIFA) quintiles**: This index relates to the area in which the survey respondent lived and may not be indicative of their individual socioeconomic status. **Equivalised income of household (quintiles)**: Standardised estimates of income using the modified OECD equivalence scale (1994) reflect the households’ relative well-being allowing for differences in household types, composition, and requirements relative to income.

Diet: Participants were asked about their usual daily consumption of vegetables and fruits from all sources, to determine whether they had met the National Health and Medical Research Council dietary guidelines (2003) [42]. **Physical activity**: Participants were asked about all their physical activity for the previous week to determine whether they had met the recommended 150 minutes of physical activity per week associated with good health, as recommended by the National Physical Activity Guidelines for Australian adults [43]. **Smoking status**: Participants were asked about their current and previous smoking status. If the participant responded that they had ever smoked, they were asked to nominate the product they smoked, the regularity, and the quantity.

### Statistical analyses

Statistical analyses described the study participants in 2 ways: (i) as a whole group and, (ii) divided into two subgroups according to Indigenous and non-Indigenous status. The variables of interest that were common to both the NNPAS and the NATSINPAS databases were identified. Data were cleaned by excluding implausible values, missing data, and outliers. The final merged dataset of NNPAS and NATSINPAS was restricted to adults aged 18 years and older who had identified the previous night’s sleep duration as ‘typical’. Assumptions for testing were confirmed, tests were two-tailed and the alpha value of 0.05 was used. Demographic and lifestyle characteristics of the sample were described using χ^2^ for categorical data and t-tests for scale data. In preliminary analyses, ANOVA with post-hoc Bonferroni corrections were used to determine the variables that significantly predict BMI and sleep duration in the whole sample. Forward stepwise regression analyses using the identified significant predictors of BMI were performed to determine the strongest predictors of BMI in the whole sample as well as in the subgroups. All analyses were completed through the ABS DataLab using IBM SPSS Statistics 24 [44].

## Results

### Demographics/Sample description

The analysis involved a total of 5,886 Australian adults: 5236 non-Indigenous and 650 Indigenous people. Table 1 describes various demographic, socioeconomic, diet, lifestyle, sleep, and BMI characteristics of the whole sample as well as the Indigenous and non-Indigenous groups.

**Table 1:** Demographic data describing whole sample and Indigenous and non-Indigenous groups.

		Whole sample N = 5886	non-Indigenous N = 5236	Indigenous N = 650		
Category	Sub-Category	N (%)	N (%)	N (%)	χ^2^ ^a^	p
**Sex**	Males	2797 (47.52)	2,526 (48.24)	271 (41.69)	9.95	0.002
	Female	3089 (52.48)	2,710 (51.76)	379 (58.31)		
**Index of Relative Socio-economic Disadvantage– 2011 (SEIFA)**	Quintile 1 (lowest)	1341 (22.78)	1,011 (19.31)	330 (50.77)	395.01	0.000
	Quintile 2	1269 (21.56)	1,109 (21.18)	160 (24.62)		
	Quintile 3	1104 (18.76)	1,029 (19.65)	75 (11.54)		
	Quintile 4	1109 (18.84)	1,045 (19.96)	64 (9.85)		
	Quintile 5 (highest)	1063 (18.06)	1,042 (19.90)	21 (3.23)		
**Labour Force Status**	Employed	3576 (60.75)	3,291 (62.85)	285 (43.85)	142.92	0.000
	Unemployed	178 (3.02)	120 (2.29)	58 (8.92)		
	Not in the labour force	2132 (36.22)	1,825 (34.85)	307 (47.23)		
**Equivalised income of household: quintiles**	Quintile 1 (lowest income)	1372 (23.31)	1,092 (20.86)	280 (43.08)	224.9	0.000
	Quintile 2	1170 (19.88)	1,008 (19.25)	162 (24.92)		
	Quintile 3	1017 (17.28)	927 (17.7)	90 (13.85)		
	Quintile 4	1177 (20.00)	1,103 (21.07)	74 (11.38)		
	Quintile 5 (highest income)	1150 (19.54)	1,106 (21.12)	44 (6.77)		
**Household type**	person living alone	1593 (27.06)	1,448 (27.65)	145 (22.31)	187.13	0.000
	couple only	1661 (28.22)	1,544 (29.49)	117 (18.0)		
	couple family with children	1627 (27.64)	1,457 (27.83)	170 (26.15)		
	One parent family with children	545 (9.26)	424 (8.1)	121 (18.62)		
	Unrelated persons aged ≥15 yrs.	136 (2.31)	126 (2.41)	10 (1.54)		
	All other households	324 (5.50)	2,37 (4.53)	87 (13.38)		
**Number of adults in household**	One	1946 (33.06)	1,711 (32.68)	235 (36.15)	5.27	0.153
	Two	3200 (54.37)	2,874 (54.89)	326 (50.15)		
	Three	534 (9.07)	469 (8.96)	65 (10.0)		
	Four or more	206 (3.50)	182 (3.48)	24 (3.69)		
**Number of children in household (0–17years)**	None	3992 (67.82)	3,660 (69.9)	332 (51.08)	147.7	0.000
	One	730 (12.40)	626 (11.96)	104 (16.0)		
	Two	743 (12.62)	634 (12.11)	109 (16.77)		
	Three	290 (4.93)	230 (4.39)	60 (9.23)		
	Four or more	131 (2.23)	86 (1.64)	45 (6.92)		
**Sleep duration categories**	< 7hrs (short sleep)	986 (16.75)	885 (16.9)	101 (15.54)	69.84	0.000
	7–9 hrs	3817 (64.85)	3,465 (66.18)	352 (54.15)		
	> 9 hours (long sleep)	1083 (18.40)	886 (16.92)	197 (30.31)		
**Body Mass Index (BMI) Categories**	Normal range BMI 18 to <25	2023 (34.37)	1,853 (35.39)	170 (26.15)	21.87	0.000
	Overweight/Obese BMI ≥ 25	3863 (65.63)	3,383 (64.61)	480 (73.85)		
**Vegetable & fruit consumption**	Met recommended guidelines	319 (5.42)	287 (5.48)	32 (4.92)	0.35	0.553
	Did not meet recommended guidelines	5567 (94.58)	4,949 (94.52)	618 (95.08)		
**physical activity last week at least 150 minutes**	Met recommended guidelines	2941 (49.97)	2,654 (50.69)	287 (44.15)	9.96	0.007
	Did not meet recommended guidelines	2908 (49.41)	2,549 (48.68)	359 (55.23)		
	Not known	37 (0.63)	33 (0.63)	4 (0.62)		
**Smoking status**	current daily	1127 (19.15)	855 (16.33)	272 (41.85)	255.46	0.000
	current weekly	76 (1.29)	66 (1.26)	10 (1.54)		
	current less than weekly	30 (0.51)	27 (0.005)	3 (0.005)		
	Ex-smoker	1971 (33.49)	1,783 (34.05)	188 (28.92)		
	Never smoked	2682 (45.57)	2,505 (47.84)	177 (27.23)		
Continuous variables		Mean (SD)	Mean (SD)	Mean (SD)	t-test value ^b^	P value
**BMI**		28.76 (6.24)	27.56 (5.5)	29.96 (6.97)	10.16	< .0001
**Age (years)**		45.82 (15.8)	49.1 (16.15)	42.55 (15.45)	9.79	< .0001
**Sleep duration (minutes)**		492.345 (90.05)	479.87 (82.67)	504.82 (97.43)	7.11	< .0001

^**a**^ χ^2^ tested differences between non-Indigenous and Indigenous groups.

^**b**^ t-tests compared means between non-Indigenous and Indigenous samples.

Overall, more females than males participated in the survey and the average age was 46 years. Forty-four (44) % of participants resided in the two most disadvantaged localities and 43% experienced the lowest household income. Almost two-thirds of participants reported that they slept for the recommended period (mean of 492 mins or approximately 8 h) on a typical night whereas the remaining participants reported either short sleep or long sleep. Almost two-thirds of participants were classed as overweight or obese, with a mean BMI of 29. Regarding diet and lifestyle factors, 95% of participants reported not meeting the recommended dietary intake of fruit and vegetables, 50% met the recommended amount of weekly physical activity and almost 20% of participants were daily smokers.

Participants were divided into two sub-groups for further analysis–Indigenous and non-Indigenous. The Indigenous group was significantly younger than the non-Indigenous group (42.6 (15.5) vs 49.1 (16.2) years of age) and was comprised of a significantly greater proportion of female participants. The Indigenous cohort was also significantly more likely to live in areas of greater socioeconomic disadvantage (40.5% vs 75.4% in Quintile 1 and 2); experience lower household income (40.1% vs 68.0% in Quintile 1 and 2); as well as higher unemployment (8.9% vs 2.3%) and higher rates of non-participation in the paid workforce (47.2% vs 34.8%) than the non-Indigenous cohort. Non-participation in the paid workforce included those who were engaged in unpaid household duties; retirees; students; and those experiencing long term infirmity [45].

The proportion of household participants living as part of a couple with children was similar for both Indigenous and non-Indigenous people, however, greater proportions of Indigenous people lived in single parent families with children (18.6% vs 8.1%) and either extended family or non-related, group-share, households than non-Indigenous people (13.4% vs 4.5%). Indigenous households were more likely than non-Indigenous households to have three or more children.

The proportions of Indigenous and non-Indigenous participants who met the recommended fruit and vegetable consumption guidelines each week were similar; however, a significantly greater proportion of Indigenous compared with non-Indigenous people did not meet the recommended 150 min of weekly physical activity (55.2% vs 48.7%), were current daily smokers (41.8% vs 16.3%) and were less likely to have never smoked (27.2% vs 47.8%).

The mean sleep duration for the Indigenous group was 504.82 (97.43) minutes (range 7.72–10.03 hours), significantly longer than the mean sleep duration of the non-Indigenous group (479.88 (82.67) minutes (range 6.60–9.34 hours)). Further examination revealed that similar proportions of participants in both groups experienced short sleep duration each night (non-Indigenous 16.9%, Indigenous 15.4%), while a significantly higher proportion of Indigenous people reported long sleep duration each night (30.3% vs 16.9%). Compared to the non-Indigenous group, the Indigenous group also had a significantly higher mean BMI (30(7) vs 27.6(5.5)).

### Predictors of sleep and BMI

Table 2 shows the predictors of sleep for the whole sample. Model 1 (adjusted-*R*^*2*^ = 0.046, *p* <0.0001) included lifestyle factors (diet, physical activity, smoking status) and sociodemographic factors (age, sex, employment status, household type, income, and SEIFA) but excluded Indigenous status. Model 2 was the same as Model 1 but with the inclusion of Indigenous status as a covariate. Both models showed similar trends: four variables significantly predicted shorter sleep duration—increased BMI, advancing age, lower household incomes, and having four or more children in the household. Longer sleep duration was significantly predicted by female sex, unemployment, and non-engagement in the workforce, and less than recommended weekly physical activity. The addition of Indigenous status in Model 2 slightly weakened the relationship between sleep duration and most covariates; the exceptions were BMI, number of children in household and lack of minimum recommended weekly physical activity, where the relationship slightly increased. Overall, accounting for Indigenous status slightly strengthened the inverse relationship between sleep duration and BMI. Model 2 also showed that Indigenous status was a significant predictor of longer sleep duration (β = 18.54, *p* <0.0001).

**Table 2:** Predictors of sleep duration.

WHOLE SAMPLE	Model 1: Predictors of Sleep duration in whole sample not accounting for Indigenous status				Model 2: Predictors of Sleep duration in whole sample accounting for Indigenous status			
	F (26, 5859) = 11.81 p<0.0001				F (27, 5858) = 12.32 p<0.0001			
Dependent Variable = SLEEP	Adjusted R-squared = 0.046				Adjusted R-squared = 0.049			
	Coeff.	p	[95% Conf. Interval]		Coeff.	p	[95% Conf. Interval]	
**Indigenous status**	NA				18.54	0.00	11.19	25.89
**Age**	-0.74	0.00	-0.91	-0.57	-0.68	0.00	-0.86	-0.51
**BMI**	-0.81	0.00	-1.19	-0.43	-0.93	0.00	-1.31	-0.55
Sex								
**Female**	12.82	0.00	8.37	17.27	12.59	0.00	8.14	17.03
Male	Reference				Reference			
**Employment Status**								
Employed	Reference				Reference			
Unemployed	22.82	0.00	9.85	35.79	20.29	0.00	7.31	33.27
Not in Labour force	22.43	0.00	16.54	28.32	21.36	0.00	15.46	27.25
**Socioeconomic status of locality**	-0.27	0.75	-1.93	1.39	0.35	0.68	-1.32	2.03
**Household type**								
Person living alone	Reference				Reference			
Couple only	-1.12	0.89	-17.65	15.41	-0.48	0.95	-16.99	16.02
Couple family with children	-10.17	0.38	-33.08	12.75	-8.36	0.47	-31.24	14.52
One parent family with children	-7.11	0.33	-21.38	7.15	-6.87	0.34	-21.11	7.36
Unrelated persons aged 15+ only	-10.97	0.33	-33.04	11.11	-9.62	0.39	-31.66	12.42
All other households	1.23	0.92	-22.15	24.60	0.40	0.97	-22.93	23.73
**Equivalised income of household**	-4.85	0.00	-6.82	-2.87	-4.56	0.00	-6.54	-2.59
**Number of adults in household**								
One	Reference				Reference			
Two	10.73	0.18	-4.83	26.28	9.87	0.21	-5.66	25.39
Three	11.11	0.27	-8.63	30.85	9.84	0.33	-9.86	29.55
Four or more	21.74	0.06	-0.45	43.93	20.98	0.06	-1.17	43.13
**Number of children 0–17 yrs in household**								
None	Reference				Reference			
One	3.06	0.59	-8.21	14.33	1.94	0.74	-9.32	13.20
Two	-8.22	0.18	-20.13	3.68	-9.67	0.11	-21.56	2.22
Three	-12.33	0.09	-26.58	1.92	-14.27	0.05	-28.51	-0.02
Four or more	-20.82	0.02	-38.70	-2.93	-24.33	0.01	-42.24	-6.43
**Fruit and vegetable intake**								
Met recommended dietary guidelines	Reference				Reference			
Did not meet recommended dietary guidelines	-1.09	0.82	-10.51	8.32	-0.94	0.85	-10.34	8.46
**Physical activity**								
Met recommended guidelines	Reference				Reference			
Did not meet recommended guidelines	6.29	0.01	1.90	10.69	6.35	0.01	1.96	10.73
Not known	-8.63	0.53	-35.60	18.35	-7.80	0.57	-34.72	19.12
**Smoking Status**								
Current daily (Reference)	Reference				Reference			
Current weekly	2.26	0.82	-17.04	21.56	4.37	0.66	-14.92	23.65
Current less than weekly	11.72	0.45	-18.41	41.85	13.45	0.38	-16.63	43.52
Ex-smoker	3.68	0.26	-2.68	10.03	5.30	0.10	-1.07	11.68
Never smoked	4.48	0.15	-1.55	10.51	6.64	0.03	0.56	12.71

Table 3 examines the predictors of BMI for the whole sample with and without the addition of sleep duration as a covariate. Model 3 (adjusted-*R*^*2*^ = 0.048, *p* <0.0001) did not account for sleep duration and indicated that significant predictors of BMI were: age, non-engagement with the workforce, socioeconomic disadvantage of the household locality, household income, miscellaneous household types (other than nuclear families and unrelated housemates), lack of minimum recommended weekly physical activity, and ex- or non-smoker status. When sleep duration was accounted for in Model 4 (adjusted-*R*^*2*^ = 0.056, *p* <0.0001), short sleep was the strongest predictor of increased BMI where a decrease in sleep duration of 1 hr less than the recommended 7 hr predicted a rise in BMI by 1 unit. Long sleep, on the other hand, was not a significant predictor of BMI. Age remained a significant predictor of BMI in Model 4, as was households of three children, lack of physical activity and ex-smoker status. Socioeconomic disadvantage of the household locality remained a significant predictor, however household income was no longer a significant predictor of BMI, nor was non-engagement with the workforce. When sleep was accounted for, compared with males, females were at significantly less risk of being overweight. Further sub-analysis showed that the predictors of BMI remained the same for the non-Indigenous group as for the whole sample, however age was the only significant predictor of BMI in the Indigenous group, where BMI increased with increased age.

**Table 3:** Predictors of BMI.

WHOLE SAMPLE	MODEL 3: Predictors of BMI not accounting for sleep duration				MODEL 4: predictors of BMI accounting for sleep duration			
	F(26, 9366) = 19.3 p<0.0001				F(26, 5859) = 14.48 p<0.0001			
Dependent Variable = BMI	Adjusted R-squared = 0.048				Adjusted R-squared = 0.056			
	Coeff.	p	[95% Conf. Interval]		Coeff.	p	[95% Conf. Interval]	
**Sleep category**								
short sleep duration < 7 hours					0.98	0.00	0.59	1.37
recommended sleep duration (7–9 hours)					reference			
long sleep duration >9 hours					0.05	0.81	-0.34	0.43
**Age**	0.05	0.00	0.04	0.06	0.05	0.01	0.04	0.06
Sex								
**Female**	-0.06	0.62	-0.31	0.18	-0.45	0.01	-0.73	-0.13
Male	reference				reference			
**Employment Status**								
Employed	reference				reference			
Unemployed	0.30	0.39	-0.38	0.98	0.67	0.13	-0.21	1.54
Not in Labour force	-0.40	0.02	-0.73	-0.08	-0.11	0.58	-0.51	0.28
**Socioeconomic status of locality**	-0.45	0.00	-0.55	-0.36	-0.48	0.00	-0.59	-0.37
**Household type**								
Person living alone	reference				reference			
Couple only	0.73	0.08	-0.10	1.57	0.51	0.37	-0.60	1.62
Couple family with children	1.08	0.06	-0.03	2.19	0.72	0.36	-0.82	2.26
One parent family with children	0.55	0.14	-0.18	1.27	0.31	0.52	-0.64	1.27
Unrelated persons aged 15+ only	0.07	0.91	-1.07	1.21	-0.37	0.63	-1.85	1.11
All other households	1.36	0.02	0.23	2.48	1.69	0.04	0.12	3.26
**Equivalised income of household**	0.13	0.02	0.02	0.24	0.11	0.09	-0.02	0.25
**Number of adults in household**								
One	reference				reference			
Two	-0.64	0.10	-1.40	0.12	-0.34	0.53	-1.38	0.71
Three	-0.89	0.06	-1.83	0.05	-0.50	0.46	-1.83	0.82
Four or more	-0.57	0.27	-1.58	0.44	-0.26	0.73	-1.75	1.23
**Number of children 0–17 yrs in household**								
None	reference				reference			
One	0.25	0.40	-0.34	0.83	0.20	0.60	-0.55	0.96
Two	-0.12	0.69	-0.72	0.48	0.20	0.63	-0.60	1.00
Three	0.47	0.20	-0.24	1.19	1.26	0.01	0.31	2.22
Four or more	0.25	0.55	-0.55	1.05	0.67	0.27	-0.53	1.87
**Fruit and vegetable intake**								
Met recommended dietary guidelines	reference				reference			
Did not meet recommended dietary guidelines	0.18	0.52	-0.36	0.71	0.21	0.51	-0.42	0.85
**Physical activity**								
Met recommended guidelines	-1.76	0.00	-2.20	-1.33	reference			
Did not meet recommended guidelines	-0.59	0.01	-1.02	-0.17	1.10	0.00	0.80	1.39
Not known	-2.12	0.01	-3.73	-0.52	0.04	0.97	-1.77	1.85
**Smoking Status**								
Current daily (reference)	reference				reference			
Current weekly	0.37	0.49	-0.67	1.41	0.33	0.62	-0.97	1.62
Current less than weekly	1.07	0.19	-0.55	2.68	0.29	0.78	-1.73	2.31
Ex-smoker	1.53	0.00	1.18	1.87	1.25	0.00	0.83	1.68
Never smoked	0.57	0.00	0.25	0.90	0.31	0.14	-0.10	0.71

Table 4 examines predictors of BMI controlling for Indigenous status. Model 5 (adjusted-*R*^*2*^ = 0.062, *p* <0.0001), did not account for sleep duration. Model 6 (adjusted-*R*^*2*^ = 0.073, p <0.0001) was the same as Model 5 but included sleep duration as a covariate. Model 5 indicated that Indigenous status was a significant predictor of BMI, with the Indigenous group being 2.61 BMI units higher than the non-Indigenous group. In Model 6 when sleep duration was controlled for in the analysis, Indigenous status remained a significant predictor of BMI. Interestingly, the inclusion of sleep duration improved the model fit (adjusted-R2 = 0.073, p <0.0001) but the relationship between Indigenous status and BMI remained significant with a slight decrease in the magnitude of the effect (β = 2.61 vs β = 2.48). A positive Sobel test (p<0.002) indicated that sleep mediated the relationship between Indigenous status and BMI.

**Table 4:** Predictors of BMI accounting for indigenous status.

WHOLE SAMPLE	MODEL 5: Predictors of BMI not accounting for sleep duration				MODEL 6: predictors of BMI accounting for sleep duration			
	F(27, 9365) = 24.16 p<0.0001				F(28, 5857) = 17.53 p<0.0001			
Dependent Variable = BMI	Adjusted R-squared = 0.062				Adjusted R-squared = 0.073			
	Coeff.	p	[95% Conf. Interval]		Coeff.	p	[95% Conf. Interval]	
**Sleep category**								
short sleep duration < 7 hours					0.97	0.00	0.58	1.35
recommended sleep duration (7–9 hours)					reference			
long sleep duration >9 hours					-0.07	0.73	-0.45	0.31
**Indigenous status**	2.61	0.00	2.18	3.04	2.48	0.00	2.00	2.97
non-Indigenous	reference				reference			
**Age**	0.06	0.00	0.05	0.07	0.06	0.00	0.05	0.07
Sex								
**Female**	-0.09	0.47	-0.34	0.15	-0.45	0.00	-0.75	-0.15
Male	reference				reference			
**Employment Status**								
Employed	reference				reference			
Unemployed	0.01	0.98	-0.67	0.68	0.33	0.46	-0.54	1.20
Not in Labour force	-0.50	0.00	-0.82	-0.17	-0.24	0.23	-0.64	0.15
**Socioeconomic status of locality**	-0.36	0.00	-0.45	-0.26	-0.39	0.00	-0.50	-0.28
**Household type**								
Person living alone	reference				reference			
Couple only	0.69	0.10	-0.13	1.52	0.59	0.30	-0.51	1.69
Couple family with children	1.07	0.06	-0.04	2.17	0.95	0.22	-0.58	2.47
One parent family with children	0.48	0.19	-0.24	1.20	0.34	0.48	-0.61	1.29
Unrelated persons aged 15+ only	0.06	0.92	-1.07	1.19	-0.19	0.80	-1.66	1.28
All other households	1.14	0.05	0.02	2.25	1.55	0.05	-0.01	3.10
**Equivalised income of household**	0.17	0.00	0.06	0.28	0.15	0.03	0.02	0.28
**Number of adults in household**								
One	reference				reference			
Two	-0.62	0.11	-1.38	0.14	-0.44	0.40	-1.48	0.59
Three	-0.87	0.07	-1.80	0.05	-0.66	0.33	-1.97	0.66
Four or more	-0.47	0.36	-1.47	0.53	-0.35	0.64	-1.83	1.13
**Number of children 0–17 yrs in household**								
None	reference				reference			
One	0.19	0.51	-0.39	0.77	0.05	0.90	-0.70	0.80
Two	-0.19	0.53	-0.79	0.40	0.00	0.99	-0.80	0.79
Three	0.33	0.36	-0.38	1.04	0.98	0.04	0.03	1.93
Four or more	-0.02	0.97	-0.81	0.78	0.18	0.76	-1.01	1.38
**Fruit and vegetable intake**								
Met recommended dietary guidelines	reference				reference			
Did not meet recommended dietary guidelines	0.18	0.52	-0.36	0.71	0.23	0.47	-0.40	0.86
**Physical activity**								
Met recommended guidelines	0.18	0.51	-0.36	0.72	reference			
Did not meet recommended guidelines	1.33	0.00	0.81	1.86	1.09	0.00	0.80	1.38
Not known	-0.07	0.94	-1.70	1.57	0.15	0.87	-1.65	1.94
**Smoking Status**								
Current daily (reference)	reference				reference			
Current weekly	0.53	0.31	-0.50	1.57	0.61	0.36	-0.68	1.89
Current less than weekly	1.27	0.12	-0.33	2.88	0.52	0.61	-1.49	2.53
Ex-smoker	1.68	0.00	1.34	2.03	1.45	0.00	1.02	1.87
Never smoked	0.81	0.00	0.49	1.14	0.59	0.00	0.19	1.00

In Model 5, non-engagement with the labour force significantly predicted BMI; however, when sleep duration was accounted for in Model 6, employment status was no longer predictive of BMI. In Model 5, sex was not a significant predictor of BMI, but when sleep was accounted for in Model 6, female sex predicted a significantly lower BMI than male sex. Model 6 also showed that short sleep duration but not long sleep, was significantly associated with increased BMI (β = 0.97, *p* <0.0001). Both models indicated that advancing age; socioeconomic disadvantage of household locality; household income; failure to meet the recommended weekly minimum 150 min of physical activity; and ex- or non-smoker status all independently predicted BMI.

## Discussion

Previous reports of the sleeping habits of Australian adults have generally agreed that both short and long sleep duration (<7 h or >9 h) are suboptimal and unhealthy [17, 46, 47]. A recent meta-analysis reported a significant association between obesity and shorter than optimal sleep duration though long sleep did not appear to significantly influence BMI [48]. Overweight/obesity has a well-established relationship with chronic illness and poor health outcomes related to cardiovascular, metabolic, and renal diseases [1], as well as numerous cancers [2], musculoskeletal disorders, and other diseases [49]. The possible influence of sleep duration on the established relationship between overweight/obesity and chronic illness is an important consideration for Indigenous Australians given the prevalence of overweight/ obesity [6] and the disparately high incidence of chronic illnesses experienced by this population [50].

The current study showed that the mean sleep duration for Indigenous Australians was significantly longer than that for non-Indigenous people. This finding echoes that of a previous studies of African Americans [51], however extremes of sleep duration have been reported for various groups [52]. The current study also found that proportionally more Indigenous people experienced longer sleep than non-Indigenous people.

Consistent with previous reports [48], and as we have previously described [21], increased BMI was independently associated with short sleep duration of less than 7 hours, but not with long sleep of greater than 9 hours. Notably, after accounting for possible confounding influences, Indigenous status was a significant predictor of BMI. Without considering the effects of sleep duration, Indigenous people were likely to be more than 2.5 BMI units higher than non-Indigenous people. This finding concurs with a recent review that showed obesity rates were significantly higher in Indigenous compared with non-Indigenous people in Australia, Canada, New Zealand, Norway, and the USA [53]. When sleep duration was accounted for, the relationship between Indigenous status and BMI remained significant, with a slight attenuation of the strength of the relationship. A positive Sobel test confirmed the partial mediating role of sleep in the relationship between Indigenous status and BMI. However, the very small drop in β coefficient indicated that the mediator effect was weak but statistically significant. Therefore, it seems that socioeconomics, lifestyle, and sleep do not account for the differences in BMI seen between Indigenous and non-Indigenous participants.

A recent Australian Institute of Health and Welfare (AIHW) report [4] identified the three main areas of concern related to the health disparities experienced by Indigenous Australians as (i) social determinants; (ii) health risk factors; and (iii) access to appropriate health services. Social determinants were determined to account for approximately 34% of the health inequity. While access to health services is not addressed by the present study, Indigenous participants in this study exhibit the characteristics of concern [4] and echo the findings of another recent Australian study [8], including lower levels of employment and income and higher rates of smoking with lower rates of physical activity present for this sample.

Relevant to the AIHW report findings, the Indigenous participants in this study tended to sleep longer, experience lower socioeconomic status and greater rates of obesity than non -Indigenous people. In addition, while short sleep duration was an independent predictor of increased BMI, it was also significantly associated with low household income and having 4 or more children in the household. Long sleep duration was significantly predicted by female sex, unemployment, and non-engagement in the workforce. However, the relationship between these social determinants and BMI were only significant in the non-Indigenous participants. Age was the only significant predictor of BMI, within the Indigenous sample. This suggests that there are different drivers of BMI in Indigenous participants, and points to structural differences within these contexts. That is, Australia’s policies for Indigenous adults differ from non-Indigenous adults in systemic ways that affect all parts of Indigenous life. Indigenous Australians experience significant socioeconomic inequality and interpersonal, institutional, and systemic racism [54]. This is important because Australia exhibits one of world’s highest levels of societal financial inequality and greatest social gradients [55], while concomitantly exhibiting elevated incidence of stress related conditions, more prevalent in people in the lower SES range [56, 57]. Indigenous Australians are over-represented in the lower SES groups of Australian society [6] and experience social insecurity, social immobility and psychological stress due to feeling disrespected, unvalued and subordinated [54, 57, 58]. This inequality renders Indigenous Australians strongly predisposed to stress related conditions [3]. Stress increases physiological inflammation, and is strongly implicated as a contributor to the development of obesity and many of the associated cardio-metabolic co-morbidities experience by Indigenous Australians [56].

### Limitations and strengths

The results of this study must be viewed in the light of several limitations. First, the use of cross-sectional data precludes the determination of causality. While statistically significant relationships were found, we cannot conclude cause and effect. In addition, statistically significant results do not always translate to clinical significance [59]. Second, self-reported data is a potential limitation due to the possibility of response bias [60]. Self-reported responses may offer under, or over, estimations of participants’ experiences due to poor inaccurate recall, misunderstanding or desire for social approval. Self-reports of fruit and vegetable intake are often prone to social approval bias [61]. Similarly, self- reported measures of physical activity may also be subject to bias. A recent study reporting international compliance with physical activity guidelines showed marked differences between subjectively self-reported and objectively measured physical activity [62]. In addition, self-reported sleep duration, although widely used may not be a reliable measure because although participants may know when they went to rest with the intention of going to sleep, they can only estimate the actual time that sleep occurred. Although not available for this study, non-invasive actigraphy is a widely used, validated tool, that offers a more objective measurement of sleep duration, despite the tendency to over-estimate sleep duration when stillness is reported as sleep [63]. Additionally, it is important to recall that self-reported sleep duration is only one aspect of the sleep picture. There are other factors related to both sleep duration and sleep quality such as sleep disturbances, comorbidities, pain, sleep apnoea, medication use, alcohol consumption, and mental health that were not examined [64].

The omission of data regarding the intake of alcohol and other substances that impact the quality and duration of sleep is another limitation of this study. It would have been useful to have included alcohol as well as prescription and non-prescription medications, and substances such as recreational or illicit drugs that act as sedatives, stimulants and psychotropics. However, such data was not available and was outside the scope of this study. It is worth noting that recent reports show that Indigenous Australians are more likely to abstain from alcohol than non-Indigenous Australians and are only slightly more likely to exceed the National Health and Medical Research Centre (NHMRC) lifetime risk guidelines for drinking than non-Indigenous people [65].

Another consideration is that although the data was collected in a robust manner through the Australian Bureau of Statistics, the small ratio of Indigenous to non-Indigenous participants in this sample limits how representative the results are in the Australian structural context. It is also important to note that a further limitation to the generalisability of this study is that Indigenous Australian people come from numerous distinct cultural groups, each with their own history, language and belief systems and who live widely varying lifestyles. Additionally, the lack of significant predictors within the Indigenous sample could be a function of lower sample size and reduced variability in the social determinants.

Australian Indigenous people have different fat distributions than non-Indigenous people with a greater propensity for abdominal adiposity and leaner limbs [66, 67]. Some authors suggest that BMI measures for Indigenous people may underestimate central abdominal obesity and hence, underestimate their risk for metabolic syndrome and chronic illness. Waist circumference and waist to hip ratio are commonly used as determinants of healthy weight because they are more precise measures of central obesity than BMI and better indicators of potential risk for the development of chronic metabolic and cardiovascular diseases [68]. In this analysis, BMI was used because it is more commonly reported in the literature and because it was highly correlated with waist measurement (r = 0.9, p< 0.01 (2-tailed)).

The ABS NATSINPAS data were collected by Indigenous Research Officers in collaboration with Indigenous organisations and stakeholders in accordance with the ethical guidelines released by the NHMRC for research with Indigenous peoples [69]. However, these guidelines also advise that Indigenous peoples’ voices are critical to all stages of the research process and the capacity building of Indigenous researchers, hence the research team included an experienced Indigenous researcher who critically reviewed and contributed an Indigenous voice to the manuscript [70].

Despite the limitations of the study, the strength of this study is that it reports one of the largest data samples of Indigenous and non-Indigenous people regarding sleep, BMI, and other socioeconomic and lifestyle factors, to date.

## Conclusion

This study found that increased BMI was independently associated with short sleep duration of less than 7 hours, but not with long sleep of greater than 9 hours. The study also showed that the mean sleep duration for Indigenous Australians was longer than that for non-Indigenous people and that Indigenous status was independently associated with increased BMI.

Previously we reported that sleep duration played a mediator role in the relationship between Indigenous status and BMI for Australian Indigenous children [16]. This study has replicated that finding for Indigenous Australian adults, whereby sleep played a weak, partial mediator role between Indigenous status and obesity. The study also indicated that socioeconomics, lifestyle, and sleep do not account for the differences in BMI seen between Indigenous and non-Indigenous participants. Future research in this area must focus on the drivers of BMI for Indigenous people in the context of their daily lives, and not as a standardised subgroup of the greater population. However, most importantly, to find meaningful ways to *Close the Gap* [71] in health inequities for Indigenous Australians, researchers, governments, and policy makers must heed the *Uluru Statement from the Heart* [72] so that Indigenous voices are heard and Australian laws and policies reflect the landmark declaration of Aboriginal and Torres Strait Islander self-determination as the key to addressing existing disempowerment and the ongoing impact of colonisation.

## Supporting information

S1 File (DOCX)
